# Thyroid Cancer Radiomics: Navigating Challenges in a Developing Landscape

**DOI:** 10.3390/cancers15245884

**Published:** 2023-12-18

**Authors:** Simone Maurea, Arnaldo Stanzione, Michele Klain

**Affiliations:** Dipartimento di Scienze Biomediche Avanzate, Università degli Studi di Napoli Federico II, 80123 Naples, Italy

In a review from 2021 by Cao et al. [1], the authors comprehensively illustrate the potential role of radiomics in well-differentiated thyroid cancer (DTC), exploring different applications, ranging from nodule characterization to locoregional DTC staging and prognostic assessments. The authors succinctly and effectively introduce the imaging modalities, both morphologic and functional, currently adopted to evaluate patients with suspected or diagnosed DTC. Thereafter, they present the concepts and definition of radiomics with specific reference to clinical applications for the detection and prediction of thyroid cancer. Briefly, radiomics is a multi-step post-processing technique that allows medical images to be mined for quantitative data [2]. The rationale of this advanced technical approach for tumor imaging assessments is that biomedical images contain quantitative diagnostic information reflecting underlying pathophysiological processes that are detectable with digital quantitative methods but not with a visual qualitative evaluation or conventional quantitative analysis. However, radiomics is methodologically complex, and there are many potential pitfalls for each component of the pipeline, which includes the use of image segmentation, feature extraction and selection and, finally, model construction (often based on machine learning algorithms) to classify or predict the tumor characteristic of interest; in particular, radiomics features may also be integrated with clinical data to build predictive models that could be helpful in clinical practice [2].

While these premises are exciting, radiomics seems to be clinically distant from routine real-world application in patients with DTC because of the methodological heterogeneity of performed studies, at least partly due to the abovementioned complexity of the radiomics pipeline [3,4], as well as the lack of external validation in the majority of the available studies reported in the literature [1]. As properly illustrated in a review by Cao et al. [1], the majority (88%) of radiomics studies differentiating malignant from benign nodules were performed using ultrasound (US) images with manual or semi-automated lesion segmentation considering a variable number of radiomics features; only in a single study by Zhou et al. [5] was external validation testing available. Similarly, the majority (71%) of radiomics studies predicting malignant prognosis in terms of the presence of lymph node and/or distant metastases, tumor progression, treatment response and gene mutation were performed using US images with a variable number of radiomics features, but manual segmentation was used in all studies; only in a single study by Jiang et al. [6] was external validation testing available. Of note, it is reasonable that the main imaging technique tested in these studies for the application of radiomics assessments to characterize thyroid nodules was US; however, although the reported accuracy values were high, the number of cases was limited, consisting of only eight investigations aiming to differentiate between benign and malignant thyroid nodules, of which one analyzed computed tomography (CT) images, and seventeen studies aiming to predict malignant prognosis, of which five used CT or magnetic resonance (MR) images. Furthermore, the wide interest of radiomics in clinical applications for the evaluation of patients with cancer, such as DTC or other tumors, observed in recent years requires the use of radiomics guidelines for research methodology to avoid technical drawbacks [7,8,9]. Moreover, in the era of “big data”, radiomics features should be assessed by integrating clinical data to build predictive models combining all sources of medical information (holomics); thus, this latter comprehensive approach should be employed and investigated in patients with DTC for whom clinical, laboratory, imaging, histopathological and genetic data are fundamental for management [10,11]. More recently, two systematic reviews were published on the topic, one focused on lymph node assessment [12] and one on nuclear medicine applications [13], both confirming the encouraging findings while highlighting similar limitations for the included radiomics studies, as previously done by Cao et al. [1]. Finally, it is worth mentioning that the first few pieces of FDA- and CE-certified dedicated software for automated thyroid nodule characterization on ultrasound have been made commercially available (https://grand-challenge.org/aiforradiology/, accessed on 30 November 2023).

In conclusion, research in the field of radiomics and DTC has made many promises, but the clinical applicability is at present still limited. Multi-center radiomics studies with external validation testing, preferably with holistic approaches and focused on robustness and generalizability, are required to establish the potential role of this advanced technique in clinical practice for the evaluation of DTC patients.
